# Genome-wide association study reveals candidate genes for traits related to meat quality in Colombian Creole hair sheep

**DOI:** 10.1007/s11250-023-03688-z

**Published:** 2023-10-12

**Authors:** Herman Alberto Revelo, Diana López-Alvarez, Yineth Alexandra Palacios, Oscar David Vergara, Moris Bustamante Yánez, Manuel Fernando Ariza, Susan Lorena Castro Molina, Yurany Ortiz Sanchez, Luz Ángela Alvarez

**Affiliations:** 1 Grupo de Investigación de Recursos Zoogenéticos, Departamento de Ciencia Animal, Facultad de Ciencias Agropecuarias, Universidad Nacional de Colombia, 763533 Palmira, Colombia; 2 Present Address: Facultad de Medicina Veterinaria y Zootecnia, Universidad San Martin Cali Colombia, Carrera 122 #23-395 del, Vía Cali-Puerto Tejada, 760022 Cali, Colombia; 3 Grupo de Investigación en Diversidad Biológica, Departamento de Ciencias Biológicas, Facultad de Ciencias Agropecuarias, Universidad Nacional de Colombia, 763533 Palmira, Colombia; 4 Grupo de Investigación en Producción Animal Tropical, Universidad de Córdoba, 14014 Córdoba, Colombia; 5 Department of Animal Production, Universidad Nacional de Colombia, 111321 Bogotá D.C, Colombia

**Keywords:** Growth, GWAS, Creole sheep, SNPs

## Abstract

Genome-wide association studies (GWAS) allow identifying genomic regions related to traits of economic importance in animals of zootechnical interest. The objective of this research was to conduct a genome-wide association study on meat quality traits using the Illumina OvineSNPs50 BeadChip array. The animals were sampled in the departments of Córdoba, Cesar, and Valle del Cauca. The genotypes obtained with the Illumina OvineSNP50 BeadChip microarray were analyzed SNP (single-nucleotide polymorphism) data to conduct a GWAS for pH and water-holding capacity (WHC) traits measured after 7 days of maturation, in the Longissimus dorsi (LD) muscle, in 167 Creole hair sheep of 12 months old belonging to Pelibuey (CHSP, *n* = 60), Ethiopian (CHSE, *n* = 44), and Sudan (CHSS, *n* = 63) breeds. The GWAS was done using a mixed linear model (MLMA) and based on the Ovis aries v3.1 genome. The CHSE showed the lowest meat juice release and, consequently, the highest water-holding capacity (WHC = 30.6 ± 0.1), suggesting that this breed has better performance in the meat industry compared with CHSS (WHC = 41.7 ± 0.1) and CHSP (WHC = 36.8 ± 0.1), since there is a relationship between WHC and juiciness. For the character pH, it was not possible to annotate genes related to meat quality, while, for the WHC, they have obtained 11 candidate genes associated (ELOVL2, ARAP2, LOC101102527, SHOC2, AIPL1, CSRNP3, IFRD, KDM8, NANS, DAPK1, IBN2, TPM2). Particularly, ELOVL2, ARAP2, IBN2, and TPM2 genes are involved in muscle contraction and fatty acid composition in sheep. In this study, we generated a baseline for GWAS related to meat quality traits in Colombian Creole hair sheep that can be used for future genomic selection plans.

**Supplementary Information:**

The online version contains supplementary material available at 10.1007/s11250-023-03688-z.

## Introduction

In Colombia, Creole hair sheep (CHS) are considered a socially important genetic resource for rural economy-based communities due to the multiple benefits that these animals provide, especially, to indigenous communities (Revelo et al. 2020). Furthermore, CHS exhibit high meat production potential given the diversity of agroecosystems and favorable conditions in the country (Revelo et al. 2020; Flórez et al. 2020).

Hair sheep are mainly raised in the tropical Caribbean region under traditional production systems that lack technology and reproductive control (Flórez et al. 2020). Additionally, a group of hair sheep called “Pelibuey” (literally, sheep with cow hair) are mainly raised in southwestern Colombia (Valle del Cauca) in association with sugarcane plantations, where they provide biological control of weeds in the rows of sugarcane grass paddocks.^1^ According to the sheep slaughter survey reported by the National Administrative Department of Statistics (*Departamento Administrativo Nacional de Estadística*) (DANE 2020), 31,978 head were slaughtered, producing 1,006,355 kg of pie and 498,679 kg of carcass meat. In the same year, the national live weight at slaughter totaled 33.1 kg ± 2.5 kg, while the weight of the carcass was 18.5 kg ± 1.2 kg with a performance of 49.5%. Sheep meat consumption is internal and regionalized. The largest volume of sheep carcass meat is commercialized in market plazas (94.5%) and has grown up to 75.0%. The consumption per capita ranges from 310 to 500 g per year (Arevalo and Correa Assmus 2013).

The increasing demand of sheep meat in the country (Mestra-Vargas et al. 2019) is generating new perspectives for livestock production. However, there are limited studies on meat quality-related traits (Navarro et al. 2015) and the parameters that define these traits (e.g., water-holding capacity and pH), causing farmers to obtain low prices that are not differentiated by the quality of the meat (Arevalo and Correa Assmus 2013). Furthermore, quality-related parameters are not implemented in genetic improvement plans since WHC and pH measurement are expensive and difficult to obtain by requiring the slaughter of the selection candidates.

Warner et al. (2010) suggest that meat quality is influenced by genetic factors such as breed and type of muscle fiber. Environmental factors such as management system, diet, age, weight at sacrifice, and *antemortem* and *postmortem* procedures also affect quality (Pearce et al. 2011; Joo et al. 2013; de Lima Júnior et al. 2016; Hervé 2013). These aspects are usually considered at each stage of the meat production chain since the consumer is not only the final recipient but also the breaking point by deciding on the final product (Hervé 2013; Leal-Gutiérrez 2013).

Currently, WHC and pH traits are used to estimate meat quality (Pearce et al. 2011; Leal-Gutiérrez 2013). According to Zhang et al. (2009), WHC defines the ability of meat to retain water during the exertion of external forces such as cutting, heating, and grounding. WHC is a highly relevant trait since it affects product performance and has important economic implications on the industry and the consumer (Leal-Gutiérrez et al. 2014; Navarro et al. 2015). On the other hand, pH is one of the main parameters used to verify meat quality since it correlates with other traits including WHC and relates to color, tenderness, juiciness, and texture (Warner et al. 2010; Pearce et al. 2011; Navarro et al. 2015).

A genome-wide association study (GWAS) is a feasible and powerful tool to discover candidate genes and loci associated with quantitative traits (Yang et al. 2011; Zhao et al. 2016; Hernández-Montiel et al. 2020). It allows not only identifying controlled phenotypes for single genes but also complex traits produced by the interaction of many genes. This methodology has been used to study diseases in sheep (White et al. 2012; Leymaster et al. 2013), characteristics associated with the carcass (Palacios 2018), variations in the number of lambs per birth in Pelibuey sheep (Hernández-Montiel et al. 2020), productivity (AI-Mamun et al. 2015), growth and meat production traits (Zhao et al. 2016), resistance to parasites (Sallé et al. 2012), among others. However, to our knowledge, there are no GWAS for meat quality traits such as WHC and pH. Therefore, the objective of this research was to use SNPs to conduct a GWAS on pH and water-holding capacity (WHC) in the *Longissimus dorsi* muscle of Pelibuey (CHS_P_), Ethiopian (CHS_E_), and Sudan (CHS_S_) Creole hair sheep.

## Materials and methods

### Ethics statement

The sample collection procedure was approved by ethics committee of Universidad Nacional de Colombia authorized the project by which this research was conducted (in minutes No.005/2019). All procedures followed the guidelines and regulations established in the Colombian code of bioethics (resolution 8430 of 1993) and Law 84/1989 regarding the protection of animals. Blood samples were collected by qualified veterinarians during their routine practice within the framework of official programs aimed at the identification, health monitoring, and parentage confirmation of the breeds and populations included in this study. The use of animals and private land in this study was approved by the respective owners.

### Animal sampling and study area

For CHS_E_, we evaluated 44 individuals (25 males and 19 females) from four farms located in the Department of Cordoba and one farm in the Department of Cesar (Table 1). The sheep were raised in extensive production systems and fed with Angleton *(Dichantium aristatum*), Brachiaria (*Brachiaria* sp.), Colosuana (*Bothriochloa pertusa*), and Guinea (*Megathyrsus maximus*) pastures. For CHS_S_, we analyzed 63 animals (42 males and 21 females) from five farms located in Cesar and one farm in Cordoba (Table 1). These animals were also raised in extensive production systems with Colosuana (*Bothriochloa pertusa*), Angleton (*Dichantium aristatum*), and Guinea (*Megathyrsus maximus*) pastures. For these farms, we were not able to obtain a family structure due to low technological levels. For CHS_P_, we evaluated 60 males from El Hatico Natural Reserve, located in the municipality of Cerrito (Valle del Cauca) (Table 1). The animals were raised in a silvopasture environment integrated with sugarcane plantations. Furthermore, the farm applies a directed breeding so we were able to obtain a family structure of 60 offspring from six fathers.

**Table 1:** List of Colombian Creole sheep breeds, geographical positions (Department, Municipality, and farm), and number of sampled animals (*N*)

Breed	Acronym	Department	Municipality	Farm	*N*
Ethiopian	CHS_E_	Córdoba	Chima	Galilea (1G)	10
			Los Córdobas	Israel (1I)	10
			San Andrés de Sotavento	Bankoovino (1B)	10
			San Pelayo	Cañabraval (1C)	10
		Cesar	Valledupar	Caracas (2C)	4
Sudan	CHS_S_	Cesar	Valledupar	San José (2SJ)	12
		Cesar	Valledupar	Villa Clara (2VC)	10
		Cesar	Valledupar	Villa Liana (2VL)	14
		Cesar	Valledupar	La Playa (2P)	8
		Cesar	Chimichanga	Los Ángeles (2A)	9
		Córdoba	Ciénaga de Oro	Un Córdoba (2U)	10
Pelibuey	CHS_P_	Valle del Cauca	Cerrito	Hatico (3H)	60

### Phenotyping for pH and WHC

**Fig. 1:**
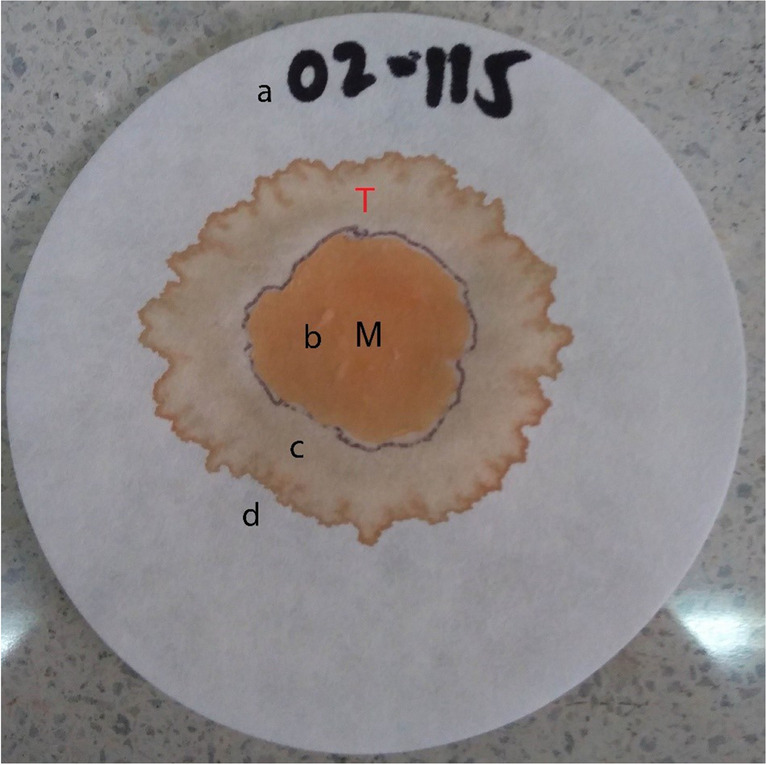
(**a**) Paper labeled with the number of the animal and the corresponding meat slice, (**b**) ground meat sample (0.3 g) on the paper after applying 2.5 kg, and (**c**) paper after the applied weight, showing two different areas; the inner ring is formed by the pressed meat (M) and the outer ring is formed by the water released by the meat (T), (**d**) delimitation of the perimeter of the two areas

In total, 167 animals were slaughtered at 12 months old at different slaughterhouses according to their origin: the animals from Cordoba were slaughtered at Frigocer Expocol S.A.S. (Cereté); those from Cesar at Echeverry Gutiérrez & CIA SC (San Juan del Cesar, La Guajira), and those from Valle del Cauca at Carnes y Derivados de Occidente S.A. (Yumbo). After slaughter and at 24-h *postmortem*, we extracted the *Longissimus dorsi* (LD) muscle from the left carcass, which was vacuum sealed and stored at 4 °C. After 7 days of maturation (aging), we measured the pH using a pH meter, with three readings per sample. Additionally, the WHC was determined according to the protocol described by Leal-Gutiérrez et al. (2014). Briefly, 20 g of the LD samples was ground for 30 s, then, approximately 0.3 g of the sample was placed on a Walkman No. 5 filter paper (Figs. s1a) and a compression force of 2.5 kg was applied for 5 min. Then, the paper was removed, revealing two areas, one formed by the pressed meat (M) and the second corresponding to the water released by the meat (T) (Fig. 1. Fig.s1b). The papers were photographed using Adobe Acrobat Professional and the two areas were measured using the measure function. The WHC is expressed as the ratio between the areas M and T, and is given by the formula: Area-M*100/Area-T.

### DNA extraction, genotyping, and quality control

A total of 10 mL of blood from each animal was collected in BD vacuum tubes (Becton Dickinson, Franklin Lakes, NJ, USA) with k3-EDTA. Genomic DNA was extracted from 167 blood samples using the QIAamp® DNA Mini Kit (QIAGEN, Hilden, Germany). DNA quantity and quality were assessed by spectrophotometry using a ColibriTM equipment (Titertek Berthold, Neulingen, Germany) and agarose gel electrophoresis using a standard protocol to evaluate sample degradation.

All animals were genotyped with the Illumina OvineSNP50 SNP chip, which allows the simultaneous characterization of up to 54,241 independent SNPs, according to the Infinium® Assay Super II Illumina® protocol for use on the HiScan®SQ System and BeadChips scanning. Quality control (QC) was performed using PLINK v1.9b5.2 (Purcell et al. 2007) to remove SNPs with a minor allele frequency (MAF) less than 0.1 (7038), a call rate less than 0.90 (3806), and a deviation from the Hardy–Weinberg equilibrium determined at *p* < 0.0001 (696). Furthermore, SNPs on sex chromosomes and the mitochondrial contig were also excluded. Finally, 42,701 SNPs remained for further analyses. The genetic structure of the population of CHS was evaluated by a multidimensional scaling analysis (MDS) based on pairwise identical-by-state (IBS) distances that were calculated and plotted using Bite (Milanesi et al. 2017) package from Bioconductor in R v3.6.0 (http://www.r-project.org).

### Statistical analyses

To evaluate the phenotypes for WHC and pH values, a descriptive analysis and an ANOVA of least squares or GLM were performed using the statistical package SAS 9.1.3 (SAS, Inc. Cary N.C). For the CHSE and CHSS breeds, the effects of different farms were considered, while for CHSP, controlled breeding was conducted on a single farm where 10 male offspring were produced per sire. For that reason, it was used two fixed models. First, model (M1) was used for CHSE and CHSS, where the following fixed effects were included: sex (male and female), farm (1B, 1C, 1G, 1I, 1U, 2A, 2C, 2P, 2SJ, 2VC, and 2VL), and lambing season (drought and rainy).

(M1)

$${\mathrm{Y}}_{\mathrm{ijkl }}=\upmu + {\mathrm{S }}_{\mathrm{i}}+{\mathrm{ F}}_{\mathrm{j}} +{\mathrm{ EP}}_{\mathrm{k}} + {(\mathrm{SF})}_{\mathrm{ij }}+ {(\mathrm{SP})}_{\mathrm{ik}} +{ (\mathrm{FP})}_{\mathrm{jk }}+ {(\mathrm{SFP})}_{\mathrm{ijk }}+{\upvarepsilon }_{\mathrm{ijkl}}$$

where *Y*_*ijkl*_ is the vector of traits WHC and pH, μ corresponds to the overall mean, *S*_*i*_ is the fixed effects of the *i*-nth sex, *F*_*j*_ is the fixed effects of the *j*-nth farm, EP_*k*_ is the fixed effects of the *k*-nth lambing season, (SF)_*ij*_ is the fixed effects of the interaction between sex and farm, (FP)_*jk*_ is the fixed effects of the interaction between sex and lambing season, (SFP)_*ijk*_ is the fixed effects of the interaction between farm and lambing season, and *Ɛ*_*ijkl*_ is the error.

Second, model (M2) included only two individuals for CHSP and the fixed effects were as follows: father sire (1, 2, 3, 4, 5, and 6) and lambing season (drought 1 and rainy 2).

(M2)

$${\mathrm{Y}}_{\mathrm{ikl}} =\upmu + {\mathrm{P}}_{\mathrm{i}}+ {\mathrm{EP}}_{\mathrm{k}}+ {(\mathrm{PEP})}_{\mathrm{ij}} +{\upvarepsilon }_{\mathrm{ikl}}$$

where *Y*_*ijkl*_ is the vector of traits WHC and pH, μ corresponds to the overall mean, *P*_*i*_ is the fixed effects of the *i*-nth father, *EP*_*k*_ is the fixed effects of the *k*-nth lambing season, (*SF*)_*ij*_ is the fixed effects of the interaction between father and lambing season, and *Ɛ*_*ikl*_ is the error.

Duncan’s pairwise comparisons test was used to determine significant differences, using S.A.S. 9.1.3.

### GWAS analysis

Before conducting the GWAS, linear models were fitted to each phenotype to identify the fixed effects models that provided the best fit (Supplementary material). A mixed linear model (MLMA) was used to control for population structure and environmental effects. Specifically, the genetics (through the sire) of the sheep and environmental effects such as, sex, farm (M1), and lambing season (M2) were controlled. Also, the model included a genomic relationship matrix (GRM) and the variance explained by the SNPs.

The model was:

$$\mathrm{y}=\upmu +\mathrm{ X\beta }+\mathrm{ Zu }+\mathrm{ e}$$

where ***y*** is the vector of phenotypes (WHC, pH), μ is the overall mean for the trait, *β* is the vector of fixed effects, **u** is the vector of random effects, ***X*** and **Z** are the design matrices mapping vectors of fixed or random effects, **G is the** genomic relationship matrix, and *Ɛ* is the vector of residual effects.

To facilitate this calculation, the genetic variance, var (G), is estimated based on the null model, *y* = μ + *u* + *e*, and is fixed while the association between each SNP and the trait is tested. In the null model, the variables indicate the following: *u* =  ~ *N* (0, *G*σ2), where *G* is the genomic relationships matrix (GRM) and σ2*u* = polygenic variance estimated by the null model. Table S3 shows the genomic association models (GWAS) using a MLMA for WHC and pH in CHSE, CHSS, and CHSP.

QQ-plots (Quantile–Quantile plots) were utilized to visualize the test statistics under the hypothesis of no association (null hypothesis) to identify if there was inflation.

The Bonferroni correction, which is a statistical technique to determine the threshold, was used. This technique divides the 0.05 significance level by the number of markers used in the GWAS (42,701 SNPs). Therefore, the significance threshold after applying the Bonferroni correction (1.17 × 10^ − 6) is − log10(*p*) ≥ 5.93, for genome-wide significance threshold. The chromosome-wide significance threshold had a value of 9.97 × 10^ − 4, which in the Manhattan plots corresponds to a value of 3.01. The Manhattan plots were generated using GWAS-tools package (Gogarten et al. 2012) in R v3.6.0 (http://www.r-project.org) to visualize the level of significance of the GWAS, the chromosomal locations, and SNPs. 

### Functional analysis

The functional information and biological processes of the mapped genes were retrieved using AnnotationHub, Mygene, and ReactomePA libraries  (Yu 2012) of Bioconductor in R v.3.6.0, (http://www.r-project.org) by assigning functions and GO terms reported in Gene Ontology (GO), Kyoto Encyclopedia of Genes (KEGG) https://www.genome.jp/kegg/), and PATHWAY. Additionally, gene networks and gene enrichment analyses were done using the Database for Annotation, Visualization and Integrated Discovery (DAVID) and its integrated databases (INTERPRO, UP-Keywords, Uniprot, SMART, and Pir-Superfamily).

## Results

### Meat quality traits — WHC and pH — in three populations of Creole hair sheep

In sheep, a normal pH ranges from 5.5 to 5.8 and is related to desirable characteristics in meat quality, such as color, shear force, and water holding capacity (Arce-Recinos et al. 2021). The average pH value of the LD in CHSE was 5.75 ± 0.1 (Table 2). We did not find significant differences between sexes or farms (Table S1a) but rather for the lambing season (*p* < 0.01). The average WHC value of the LD in CHSE was 30.63% ± 10.9 and no significant differences were observed between sexes (Table 2, Table S1b). However, we found significant differences between farms (*p* < 0.05), specifically, for farm 2C. This farm is the only one located in the Department of Cesar (Table 1) in a Tropical Very Dry Forest according to Holdridge’s life zones (Palacios 2018). Furthermore, we could not evaluate 10 animals in farm 2C since it applies a low-technology production system that limited the possibility of conducting breeding control.

**Table 2:** Comparison of mean values of meat quality-related traits, WHC and pH, according to sex, farm, and lambing season in Ethiopian Creole hair sheep CHS_E_

Sources of variance	Factor	*N*	WHC (%)	pH
Sex	Female	19	28.91 ± 8.2^a^	5.72 ± 0.1^a^
	Male	25	31.93 ± 12.5^a^	5.78 ± 0.1^a^
Farm	1G	10	28.86 ± 8.8^b*^	5.79 ± 0.07^a^
	1B	10	25.52 ± 6.2^b*^	5.70 ± 0.1^a^
	1C	10	27.75 ± 6.1^b*^	5.72 ± 0.07^a^
	1I	10	34.10 ± 14.9^b*^	5.81 ± 0.15^a^
	2C	4	46.32 ± 8.76^a*^	5.71 ± 0.05^a^
Lambing season	Rainy	19	32.16 ± 12.22^a^	5.8 ± 0.1^a*^
	Drought	25	29.46 ± 9.90^a^	5.71 ± 0.1^b*^
	Total mean	44	30.63 ± 10.9	5.75 ± 0.1

Mean + s.d. of variables. Superscripts denote Duncan pairwise comparisons between sources of variance; means with the same letter do not differ significantly (**p < 0.001; *p < 0.05); *N*, number of observations

The average pH value of the LD in CHSS was 5.7 ± 0.1 (Table 3). Furthermore, we observed significant differences between farms (*p* < 0.01) and for the interaction farm*lambing season (*p* < 0.05, Table S1c). Moreover, for WHC of the LD in CHSS, the average value was 41.69% ± 16.3 and we determined significant differences between farms (*p* < 0.01) and the interaction farm*lambing season (*p* < 0.01, Table S1d). Farm 1U showed the lowest WHC (Table 3) and significantly differed from the other farms. This farm is located in the Department of Cordoba (Table 1) in a Tropical Dry Forest according to Holdridge’s life zones (Palacios 2018).

**Table 3:** Comparison of mean values of meat quality-related traits, WHC and pH, according to sex, farm, and lambing season in Sudan Creole hair sheep CHS_S_

Sources of variance	Factor	*N*	WHC (%)	pH
Sex	Female	21	38.65 ± 15^a^	5.73 ± 0.1^a^
	Male	42	43.21 ± 16.5^a^	5,75 ± 0.1^a^
Farm	1U	10	25.06 ± 6.1^b*^	5.69 ± 0.13^b*^
	2SJ	12	49.27 ± 27^a*^	5.71 ± 0.1^b*^
	2VC	10	43.88 ± 16^a*^	5.81 ± 0.05^a*^
	2A	9	44.24 ± 8.7^a*^	5.71 ± 0.1^b*^
	2VL	14	40.90 ± 4.9^a*^	5.71 ± 0.1^b*^
	2P	8	46.90 ± 8.1^a*^	5.85 ± 0.09^a*^
Lambing season	Rainy	17	46.6 ± 25^a^	5.77 ± 0.1^a^
	Drought	46	39.8 ± 11^a^	5.73 ± 0.01^a^
	Total mean	63	41.69 ± 16.3	5.74 ± 0.1

Mean + s.d. of variables. Superscripts denote Duncan pairwise comparisons between sources of variance; means with the same letter do not differ significantly (**p < 0.001; *p < 0.05); *N*, number of observations

Finally, the average pH value of the LD in CHSP was 5.74 ± 0.1 (Table 4). We determined significant differences between fathers (*p* < 0.001, Table S1e); accordingly, the lowest values were observed for individuals from fathers 1 and 2. Moreover, for WHC, ram lambs showed an average value of 36.8% ± 8.8 (Table 4). We found significant differences between fathers (*p* < 0.001, Table S1f), and the highest values were obtained for fathers 3 (38.47% ± 9.13), 4 (41.64% ± 7), 5 (41.17% ± 7.25), and 6 (42.54% ± 7.4).

**Table 4:** Comparison of mean values of meat quality-related traits, WHC and pH, according to father and lambing season in Pelibuey Creole hair sheep CHS_P_

Sources of variance	Factor	*N*	WHC (%)	pH
Father	1	10	28.47 ± 3.96^b**^	5.68 ± 0.05^b**^
	2	10	28.49 ± 3.38^b**^	5.62 ± 0.1^b**^
	3	10	38.47 ± 9.13^a**^	5.79 ± 0.1^a**^
	4	10	41.64 ± 7^a**^	5.78 ± 0.1^a**^
	5	10	41.17 ± 7.25^a**^	5.79 ± 0.1^a**^
	6	10	42.54 ± 7.4^a**^	5.78 ± 0.1^a**^
Lambing season	Rainy	18	38.06 ± 7.7^a^	5.74 ± 0.1^a^
	Drought	42	36.26 ± 9.37^a^	5.7 ± 0.1^a^
	Total mean	60	36.8 ± 8.8	5.74 ± 0.1

Mean + s.d. of variables. Superscripts denote Duncan pairwise comparisons between sources of variance; means with the same letter do not differ significantly (**p < 0.001; *p < 0.05); *N*, number of observations

### Population structure

**Fig. 2:**
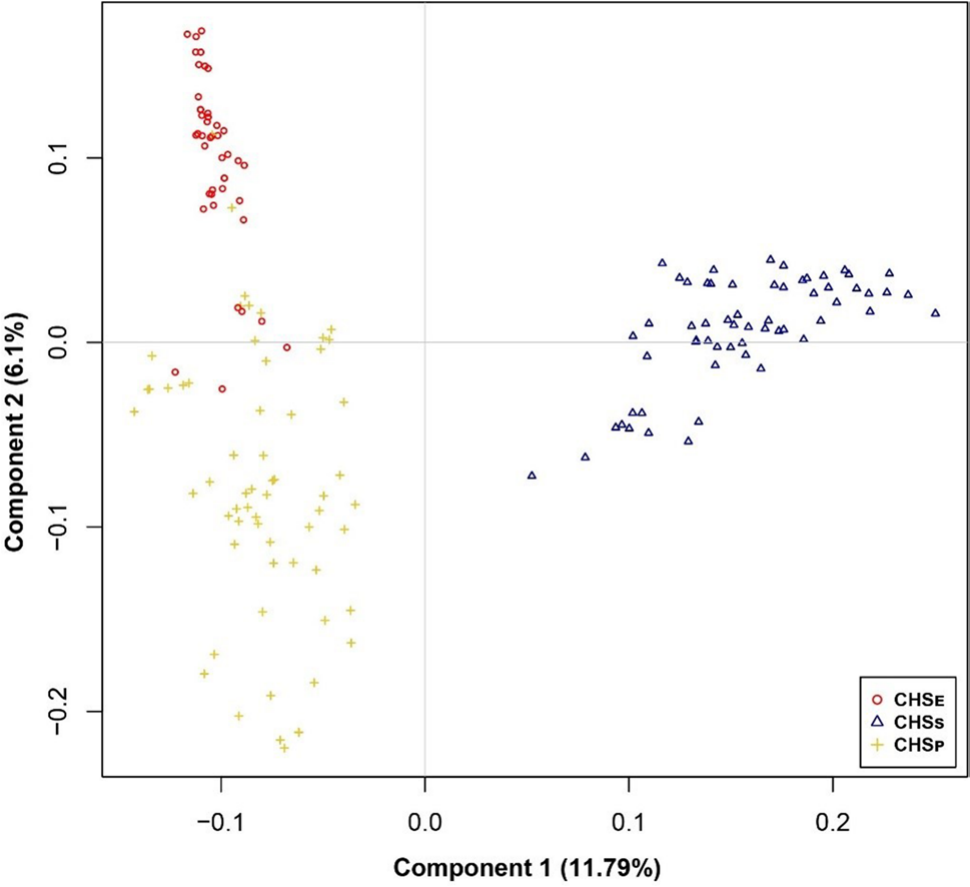
Two-dimensional PCA plot of 44 individuals of CHS_E_, 63 of CHS_S_, and 60 of CHSP based on 42,701 SNPs

The first two components of the bidimensional PCA plot explained 11.8% and 6.1% of the total variance, respectively. Furthermore, we observed a separation between CHS_P_ (blue) vs CHS_E_ (red) and CHS_S_ (yellow) along the first axis, and a secondary split of CHS_S_ vs CHS_E_ along the second axis (Fig. 2). However, a slight overlap was observed between individuals of CHS_S_ and CHS_E_, caused by a group of Sudan Blanco sheep (*n* = 8) that was sampled at La Playa farm in Valledupar. This group of sheep shares a common genetic background with CHS_E_ (*n* = 4), which were sampled at Caracas farm in Valledupar. Consequently, the overlap indicates that Sudan Blanco sheep are a mix between CHS_E_ and CHS_S_. We observed population stratification due to the presence of the three breeds from the different farms with contrasting genetic backgrounds. Individual kinship and population stratification in GWAS are the main causes of false positive correlations; therefore, we performed a GWAS per breed to identify candidate SNPs and genes.

### Genome-wide association study (GWAS) of meat quality traits — WHC and pH

**Fig. 3:**
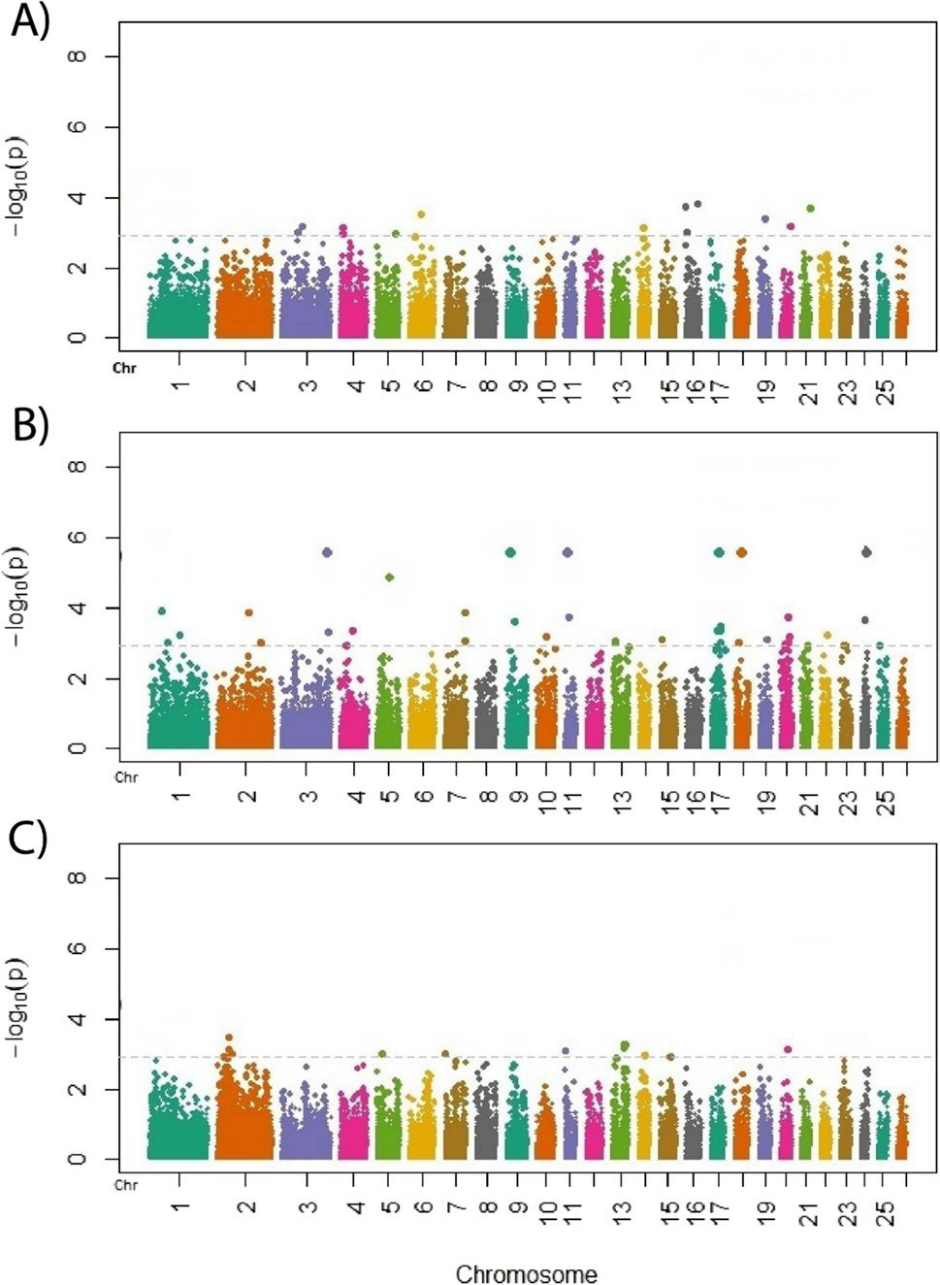
Manhattan plots of WHC for three Colombian Creole-hair sheep breeds: (**A**) CHS_E_; (**B**); CHS_S_; (**C**) CHS_P._ The grey horizontal lines in the Manhattan plots indicate the significance thresholds (*p* < 0.001). The *x*-axis shows the physical positions of the SNPs per chromosome based on the *Ovis aries* v3.1. genome assembly

A GWAS for each of the three breeds was tested using mixed linear models (MLMA) with fixed effects. The criterion applied to select the genomic association model was based on previous results of an ANOVA on phenotypic data (Table S1a, b, c, d, e, f). The GWAS of pH did not show significantly associated SNPs. On the other hand, the GWAS of WHC for CHSE showed 12 significant SNPs located on eight chromosomes (3, 4, 6, 14, 16, 19, 20, and 21) and eight genes (Table 5). Furthermore, 25% of these SNPs were found in intergenic regions, while 66.6% were in introns and 8.3% in exons (Table 5). The Manhattan plot of WHC for CHSE is shown in Fig. 3A. For CHSS, we found 33 SNPs significantly associated with WHC (*p* < 0.001, Table 6), located on 16 chromosomes (1–7, 9, 11, 13, 15, 17–20, 22, and 24) and 28 genes (Table 6). The SNPs were distributed throughout intergenic regions (33.3%), introns (60.6%), promotors (3%), and exons (3%). The Manhattan plot of WHC for CHSS is shown in Fig. 3B. Finally, for CHSP, we identified nine SNPs significantly associated with WHC (*p* < 0.001, Table 7), located on five chromosomes (2, 5, 11, 13, and 20) and eight genes (Table 7). Furthermore, 44.44% of the SNPs were found in intergenic regions, 33.33% in introns, and 11.11% in promoters. The Manhattan plot of WHC for CHSP is shown in Fig. 3C.

**Table 5:** Details of SNPs significantly associated with WHC for CHS_E_ using MLMA and nearest candidate genes

SNP	Chromosome	Position	− log10(*p*-value)	Gene	Gene description	Annotation
OAR16_57770340.1	16	53058133	1.55E + 01	CDH18	cadherin 18	Intron
OAR16_57803053.1	16	53091017	1.55E + 01	CDH18	cadherin 18	Intron
s03399.1	16	674786	1.73E + 01	SLIT3	slit guidance ligand 3	Intron
OAR21_54114533.1	21	48838644	1.98E + 01	NA	–	Distal Intergenic
s36343.1	6	55461883	2.86E + 01	**ARAP2**	ArfGAP with RhoGAP domain, ankyrin repeat and PH domain 2	Exon
s35737.1	19	30792252	4.08E + 01	NA	–	Distal Intergenic
OAR3_96465036.1	3	90809192	6.35E + 01	LTBP1	latent transforming growth factor beta binding protein 1	Intron
OAR20_48109414.1	20	44188831	6.40E + 01	**ELOVL2**	ELOVL fatty acid elongase 2	Intron
OAR14_29971688.1	14	28809538	7.16E + 01	CDH8	cadherin 8	Intron
OAR4_15640071.1	4	15399962	7.41E + 01	COL28A1	collagen type XXVIII alpha 1 chain	Intron
OAR16_7661219.1	16	7207579	9.46E + 01	NA	–	Distal Intergenic
s67263.1	3	75480558	9.52E + 01	LOC105606945	–	Intron

Genes within candidate regions were annotated based on the *Ovis aries* v.3.1 genome assembly; those in bold are the most promising candidate genes based on their effect on meat quality or identified as harboring significant SNP associated with sheep meat quality

**Table 6:** Details of SNPs significantly associated with WHC for CHS_S_ using MLMA and nearest candidate genes

SNP	Chromosome	Position	− log10(*p*-value)	Gene	Gene description	Annotation
OAR3_218139642.1	3	202690,985	2.50E − 02	ENSOARG00000026051	–	Intron
s10070.1	9	19268400	2.50E − 02	SNORA25	–	Distal Intergenic
s50062.1	11	16487540	2.50E − 02	ASIC2	acid sensing ion channel subunit 2	Intron
OAR17_39271762.1	17	36371262	2.50E − 02	FSTL5	follistatin like 5	Intron
s17049.1	18	28968415	2.50E − 02	OTUD7A	OTU deubiquitinase 7A	Intron
OAR24_27265846.1	24	24776080	2.50E − 02	KDM8	lysine demethylase 8	Intron
OAR5_68161647_X.1	5	62000366	1.38E − 01	NA	–	Distal Intergenic
s10696.1	1	52719709	1.28E + 01	ST6GALNAC5	–	Distal Intergenic
OAR2_151938109.1	2	142963793	1.30E + 01	CSRNP3	cysteine and serine rich nuclear protein 3	Distal Intergenic
OAR7_100406183.1	7	92215284	1.30E + 01	NA	–	Distal Intergenic
OAR11_26036763.1	11	25104680	1.85E + 01	**AIPL1**	aryl hydrocarbon receptor interacting protein like 1	Distal Intergenic
OAR20_39711404.1	20	36261532	1.86E + 01	CDKAL1	CDK5 regulatory subunit associated protein 1 like 1	Intron
OAR24_21411103.1	24	19579841	2.31E + 00	LOC101114079	ATP-binding cassette sub-family A member 3-like	Intron
OAR9_37599748.1	9	35709024	2.35E + 01	XKR4	–	Intron
OAR17_49866586.1	17	45793784	3.12E + 01	ADGRD1	adhesion G protein-coupled receptor D1	Intron
s43108.1	17	47855868	4.18E + 01	GLT1D1	glycosyltransferase 1 domain containing 1	Intron
OAR4_59276586.1	4	56032346	4.42E + 00	ZNF277	zinc finger protein 277	Intron
OAR17_28971772.1	17	26404750	4.45E + 01	NA	–	Distal Intergenic
s65074.1	3	213086338	4.98E − 01	BCL2L13	–	Intron
OAR22_34955585_X.1	22	30443185	5.71E + 01	SHOC2	SHOC2 leucine rich repeat scaffold protein	Intron
OAR1_147233859.1	1	136261672	5.72E + 01	NA	–	Distal Intergenic
s52527.1	20	39053345	6.60E + 01	LOC105603846	–	Intron
OAR10_48194802.1	10	47420556	6.83E + 01	LOC101122287	–	Intron
OAR19_39736257.1	19	37888856	7,56E + 01	SYNPR	synaptoporin	Intron
OAR15_5833133.1	15	6314820	7.73E + 01	CEP126	centrosomal protein 126	Distal Intergenic
s55275.1	13	11004130	8.41E + 01	**PRKCQ**	protein kinase C theta	Intron
OAR7_104475739.1	7	95912557	8.55E − 4	NA	–	Distal Intergenic
OAR2_204712172.1	2	193176,635	9.23E + 01	LOC105610065	–	Intron
s10999.1	17	45411347	9.35E + 01	ENSOARG00000025678	–	Exon
OAR1_85261298.1	1	79964281	9.52E + 01	RNPC3	RNA binding region (RNP1, RRM) containing 3	Promoter
s07011.1	13	11383479	9.91E + 01	SFMBT2	Scm like with four mbt domains 2	Distal Intergenic
s24860.1	18	13745532	9.93E + 01	ST8SIA2	ST8 alpha-N-acetyl-neuraminide alpha-2,8-sialyltransferase 2	Intron
s69516.1	20	29281487	9.98E + 00	ZSCAN23	–	Intron

Genes within candidate regions were annotated based on the *Ovis aries* v.3.1 genome assembly; those in bold are the most promising candidate genes based on their effect on meat quality or identified as harboring significant SNP associated with sheep meat quality

**Table 7:** Details of SNPs significantly associated with WHC for CHS_P_ using MLMA and nearest candidate genes

SNP	Chromosome	Position	− log10(*p*-value)	Gene	Gene description	Annotation
OAR2_56383269.1	2	52536647	3.38E + 01	**TPM2**	tropomyosin 2	Promoter (< = 1 kb)
OAR13_65478113.1	13	60250874	5.27E + 01	NA	–	Distal Intergenic
OAR13_69620023.1	13	64546634	5.44E + 01	PHF20	PHD finger protein 20	Intron
OAR13_63071015.1	13	57933199	6.42E + 01	LOC105606211	–	Distal Intergenic
s33429.1	20	32872334	6.91E + 01	LOC105603826	–	Distal Intergenic
s54820.1	2	49365215	7.42E + 01	NANS	N-acetylneuraminate synthase	Promoter (2-3 kb)
OAR11_3378385.1	11	4045712	7.76E + 01	ENSOARG00000026349	–	Distal Intergenic
s11926.1	2	31911076	9.294E + 01	**DAPK1**	Death Associated Protein Kinase 1	Intron
s16895.1	5	23139577	9.77E + 00	**FBN2**	fibrillin 2	Intron

Genes within candidate regions were annotated based on the *Ovis aries* v.3.1 genome assembly; those in bold are the most promising candidate genes based on their effect on meat quality or identified as harboring significant SNP associated with sheep meat quality

### Gene ontology (GO) and KEGG gene enrichment analysis for WHC in Colombian Creole hair sheep breeds

For CHS_E_, 20 GO terms were assigned to the *ARAP2* gene (Table S2a). Additionally, we identified three terms for the *ELOVL2* gene based on KEGG functional assignments (Table S2a). Regarding CHS_S_, 110 GO terms were assigned to five genes (Table S2b). Most terms were assigned to the *PRKCQ* gene (Table S2b), followed by *AIPL1*, *SHOC2*, and LOC101102527. The functional KEGG annotation revealed seven terms related to *PRKCQ* and one term to LOC101102527 (Table S2b). Additionally, a pathway enrichment analysis using DAVID significantly associated the term “nucleus” (*p* = 5.7E − 2) to four genes, namely *AIPL1*, *CSRNP3*, *IFRD1*, and *KDM8*. Finally, for CHS_P_, 50 GO terms were associated with two genes (Table S2c), mostly *FBN2*, followed by *TPM2*. The KEGG annotation showed six terms associated with *DAPK1*, *TPM2*, and *NANS*.

## Discussion

The water-holding capacity is possibly one of the most important quality traits of raw meat products (Huff-Lonergan and Lonergan 2005). Therefore, new methodologies should be implemented to improve economically important traits in the meat industry (Leal-Gutiérrez et al. 2014). We also evaluated the pH of the LD muscle since it is directly related to WHC (Pearce et al. 2011; Torrescano et al. 2009). Most water in muscle is retained in the myofibrils (Huff-Lonergan and Lonergan 2005); therefore, an accelerated decrease in pH and consequent reduction in WHC generate the Pale, Soft, and Exudative (PSE) condition of meat (Warner et al. 2010). On the other hand, high pH values result from a slow death since chronic stress for a long period before slaughter reduces glycogen reserves in the muscle and causes a low lactic acid content. For this reason, a higher pH at 24-h *postmortem* (6.0 to 6.8) compared to normal meat (5.4 to 5.9) turns the meat dark, firm, and dry (DFD) with a high WHC (Pearce et al. 2011), These characteristics cause dark cuts and the meat will likely be rejected by the consumer, given the impression that is not appetible or comes from old animals (Leal-Gutiérrez et al. 2014).

In this study, the pH values (CHS_E_ = 5.75, CHS_S_ = 5.7, CHS_P_ = 5.74) indicated that the meats were red, firm, and non-exudative (RFN), which are optimal meat quality parameters (Gallo and Tadich 2008). This good meat quality was attributed to relevant environmental factors, such as animal manipulation and carcass management for 24 h before and after slaughter according to the established protocols. These findings demonstrate that an adequate management before and after slaughter is essential to obtain good quality meat.

The WHC is expressed as a percentage and is related to the amount of juice released by compression or manipulation; therefore, greater juice release indicates lower liquid retention and, consequently, lower WHC (Leal-Gutiérrez et al. 2014; Navarro et al. 2015). The amount and location of water in meat can vary depending on several factors related to the muscle tissue Pearce et al. 2011). Generally, in sheep, the water-holding capacity decreases with age so adult sheep meat releases a higher percentage of juice than lamb meat. However, scientific reports on the WHC of sheep meat are varied. For example, Pérez et al. (2006) indicated low values for lactating lamb of Suffolk Down × German Precocious Merino slaughtered at live weights of 10 and 15 kg, reporting a WHC of 14.5% in females and 12.5% in males. On the other hand, a WHC of 42.4 ± 2.3 (*p* ≤ 0.05) was reported for individuals of Pelibuey × Katadin × BlackBell that were slaughtered at an average weight of 40 kg at 10 months old (Ramírez-Bribiesca et al. 2007). We report a WHC at 7-day *postmortem* of 41.69% for CHS_S_, 30.63% for CHS_E_, and 36.8% for CHS_P_ slaughtered at 12 months old. Furthermore, we found that CHS_E_ showed lower juice release and, consequently, higher WHC, suggesting that sheep of this breed can exhibit better performance in the meat industry compared with CHS_S_ and CHS_P_.

We did not find significant differences in the WHC of CHS_E_ and CHS_S_ between sexes, probably because these animals were managed under extensive grazing. These results agree with Expósito et al. (2003), who did not report differences in the WHC of the *Longissimus dorsi* muscle between sexes in Segureña sheep and attributed these findings to similarities in intramuscular fat.

Moreover, we determined significant differences in WHC for CHS_E_ and CHS_S_ between farms, which can be attributed to the geographic location of the farms (Table 2, Table 3). The farms in the Department of Cordoba are located in a Tropical Dry Forest, while the farms in Cesar are found in a Tropical Very Dry Forest.

For CHS_P_, we determined significant differences between fathers (Table 4) and these findings could be associated with the genetic traits of the father. Conversely, no differences were observed between lambing season (drought and rainy), which is possibly due to silvopasture at El Hatico natural reserve that guarantees the supply of high-quality forage year-round. In this farm, the animals graze on the sugarcane paddocks and are supplemented with balanced diets. Similarly, Ramírez-Bribiesca et al. (2007) reported a WHC of 42.4 ± 2.3 (*p* ≤ 0.05) in Pelibuey × Katadin × BlackBell sheep raised in an intensive system and slaughtered at an average weight of 40 kg at 10 months old. Moreover, Frías et al. (2011) reported a WHC of 12.8 ± 0.16 in Pelibuey with Katahdin and Dorper lambs fed with grass and supplemented with fermented sugarcane, which were sacrificed at 20 kg of weight.

Regarding pH, we did not find significant differences between sexes for CHS_E_ and CHS_S_. These results agree with Rodríguez et al. (2011), who reported a pH of 5.65 and 5.61 at 24-h *postmortem* in the *Longissimus lumborum* and *semimembranosus* muscles in Assaf and Merino × Assaf sheep. Moreover, the effect of farm on pH was significant for CHS_S_ and the highest values were observed in farms 2VC (5.81 ± 0.05) and 2P (5.85 ± 0.09). Conversely, we did not find significant effects of farm on pH for CHS_E_. For this breed, the pH values ranged from 5.7 to 5.8 and corresponded to the optimal values for meat quality. These findings are attributed to an adequate management of the animals before and after slaughter, according to standardized protocols.

### SNP markers associated with WHC, gene ontology, and metabolic pathways

For CHS_E_, we found two SNPs potentially associated with WHC. The first SNP, OAR20_48109414.1, is located in the *ELOVL2* (fatty acid elongation) gene, which encodes the enzyme that catalyzes the synthesis of long-chain polyunsaturated fatty acids (C20- and C22-PUFA), acting specifically on polyunsaturated acyl-CoA and with greater activity towards acyl-CoA C20: 4 (n-6). This enzyme participates in the production of polyunsaturated VLCFA of different chain lengths that are involved in several biological processes as membrane lipid precursors and lipid mediators (UniProt Consortium 2015). Similarly, Bolormaa et al. (2016) found SNPs strongly associated with polyunsaturated fatty acid concentrations, such as OAR20_44.1, located at 100 Kb from the *ELOVL2* gene, in a GWAS of 56 traits (e.g., weight, fat, muscling, tenderness, meat color, pH level, and fatty acid profile) in 10,613 in nine sheep breeds (Merino, Poll Dorset, Border Leicester, Suffolk, White Suffolk, Texel, Corriedale, Coopworth, and various crosses). *ELOVL2*, along with *PNPLA3* and *FADS2* genes, participate in fatty acid synthesis and metabolism. Moreover, the *EVOVL6* gene was found as a candidate affecting the composition of palmitic and palmitoleic fatty acids (Bolormaa et al. 2016). These two genes were also found in the dorsal fat of pigs Corominas et al. 2013).

The second candidate SNP found in CHS_E_ is OAR6_ s36343.1, which is located in the *ARAP2* gene and encodes the Arf-GAP with Rho-GAP domain, ANK repeat, and PH domain-containing protein 2. This protein modulates actin cytoskeleton remodeling by regulating ARF and RHO family members and is activated by binding to phosphatidylinositol 3,4,5-trisphosphate (PtdIns 3,4,5 P3) (UniProt Consortium 2015). Bolormaa et al. (2016) also identified *ARAP2* as a candidate gene associated with meat color and concentration of polyunsaturated fatty acids (omega-3 and omega-6).

For CHS_S_, we propose eight candidate SNPs, including five that are associated with WHC, namely s55275.1 located in *PRKCQ*; OAR11_26036763.1 in *AIPL1*; OAR2_151938109.1 in *CSRNP3*; OAR4_59276586.1 in *IFRD1*; and OAR24_27265846.1 in *KDM8*. Altogether, these genes participate in the metabolic pathway of “nucleus processes.” Particularly, *PRKCQ* encodes a protein kinase C theta type, which belongs to the family of serine/threonine-specific kinase proteins that are activated by calcium and diacylglycerol. These protein family members phosphorylate a wide variety of protein targets and are involved in several cell signaling pathways (UniProt Consortium 2015). We found that this gene is associated with several terms involving immune-related functions, as well as with the term adipocytokine signaling pathway (oas04920) (see Supplementary Table S2c). The *IFRD1* gene encodes the interferon-related developmental regulator 1 and can function as a transcriptional coactivator that controls the growth and differentiation of specific cell types during embryonic development and tissue regeneration (Stelzer et al. 2016). In sheep, this gene is associated with myoblast growth and muscle differentiation (Cheng et al. 2020). Moreover, *KDM8* encodes a bifunctional enzyme that acts as an endopeptidase and a 2-oxoglutarate-dependent monooxygenase (Liu et al. 2016). The photoreceptor/pineal-expressed gene, *AIPL1*, encodes the arylhydrocarbon interacting protein-like 1 and is found in the candidate LCA4 region. This encoded protein contains three tetratricopeptide motifs, compatible with nuclear transport or chaperone activity (Stelzer et al. 2016). Finally, SNP OAR24_21411103.1 located in LOC101102527, SNP OAR22_34955585_X.1 in SHOC2, and SNP OAR2_151938109.1 in CSRNP3 are not reported in the literature so their functions related to WHC are unknown.

For CHS_P_, we found nine SNPs, including four associated with WHS. First, SNP s11926.1 is located in the *DAPK1* gene that encodes a calcium-dependent threonine kinase protein. This protein is involved in several cellular signaling pathways that activate cell survival, apoptosis, and autophagy; specifically, regulating type I apoptotic and type II autophagic cell death signals, depending on the cellular setting (UniProt Consortium 2015). In this study, this gene was found associated with the term autophagy — animal (oas04140) (Table S2c). Singh et al. (2016) found it related to programmed cell death or apoptosis. Second, SNP s16895.1 is found in *FBN2*, which encodes the fibrilin-2 protein involved in the formation of a binding network with other proteins to produce filiform filaments called microfibrils in the muscle. The microfibrils are a component of elastic fibers that allow the skin, ligaments, and blood vessels to stretch (Frédéric et al. 2009). Researchers have suggested that fibrilin-2 plays a role in the direction of assembly of elastic fibers during embryonic development. Microfibrils also contribute to the establishment of more rigid tissues that support the crystalline lens, nerves, and muscles; furthermore, they contain the transforming growth factor beta (TGF-β) that inactivates them. When the microfibrils are released, TGF-β is activated and affects tissue growth and repairment throughout the organism (Frédéric et al. 2009; Ramirez & Dietz 2007). On the other hand, SNP OAR2_56383269.1 is located in the *TPM2* gene that regulates the production of beta (β)-tropomyosin, which regulates muscle fiber tension (muscular contraction) by controlling myosin and actin binding. In non-muscle cells, tropomyosin proteins play a role in controlling cell shape (Tajsharghi et al. 2007; Ochala et al. 2007). β-tropomyosin is mainly found in skeletal muscles, which are used for movement. This protein helps to regulate muscle contraction by interacting with other muscle proteins, particularly, myosin and actin. These interactions are essential to stabilize and maintain sarcomeres within muscle cells (Tajsharghi et al. 2007; Ochala et al. 2007). Sarcomeres are the basic units of muscle contraction and they are composed of proteins that exert the mechanical force needed for muscle contraction. Noce et al. (2018) determined that *TPM2*, as well as *ACTA1*, *MYLPF*, *MYH2*, *MYH7*, *TPM2*, and *TTN*, encode myofibril proteins involved in muscle contraction in the *Longissimus dorsi* muscle in autochthonous Spanish sheep breeds (Canaria de Pelo, Roja Mallorquina, Gallega, Xisqueta, and Ripollesa). Regarding metabolic pathways associated with striated muscle contraction, we found that gluconeogenesis, glycolysis, the citric acid cycle, and the electron transport chain were enriched. Finally, SNP OAR2_56383269.1 is located in the *NANS* gene that encodes an enzyme involved in sialic acid biosynthesis. This protein uses N-Acetyl-mannosamine 6-phosphate and mannose 6-phosphate as substrates to produce phosphorylated forms of N-acetylneuraminic acid (Neu5Ac) and 2-keto-3-deoxy-D-glycero-D-galacto-nononic acid (KDN) (Stelzer et al. 2016).

## Conclusion

We did not find SNPs significantly associated with pH probably since the measurements were taken at 7-day *postmortem*, when most biochemical and physical processes are stable. Therefore, this trait must be evaluated at the moment of slaughter and within 3-h *postmortem* to determine the velocity at which the pH decreases.

We generated a baseline for sheep farmers who seek to improve meat and carcass quality based on a genome-wide association study of meat quality traits in Colombian Creole hair sheep. We found that the LD muscle of CHS_E_ releases fewer juice and, consequently, has greater WHC, suggesting that these animals will exhibit better performance in the meat industry. Overall, we identified candidate genes that are promising for genomic selection (*ELOVL2*, *ARAP2*, *FBN2*, and *TPM2*) and are involved in muscle contraction, metabolism, and fatty acid composition.

### Supplementary Information

Below is the link to the electronic supplementary material.

Supplementary file1 (XLSX 12 KB)

Supplementary file2 (XLSX 32 KB)

Supplementary file3 (XLSX 14 KB)

Supplementary file4 (DOCX 1359 KB)

Supplementary file5 (DOCX 51 KB)

## Data Availability

The data sets generated or analyzed during the current study, or both, are available from the corresponding author upon reasonable request.
