# Supplementary material for: Genome-wide association study reveals candidate genes for traits related to meat quality in Colombian Creole hair sheep
Source: Trop Anim Health Prod. 2023 Oct 12;55(6):357. doi: 10.1007/s11250-023-03688-z (PMC10570192; doi:10.1007/s11250-023-03688-z)
Supplement: Supplementary file 4 — Supplementary file4 (DOCX 1359 KB) [file 11250_2023_3688_MOESM4_ESM.docx]

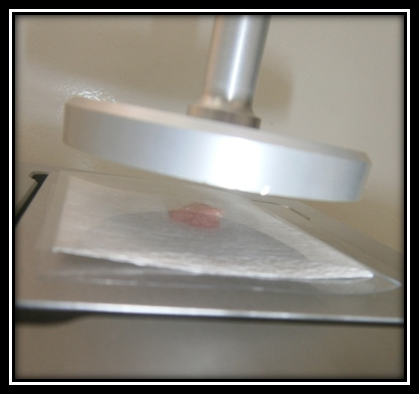

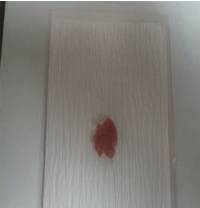


**A**

**B**

**Supplementary Figure S1a**: Briefly, 20 g of the LD samples were ground for 30 seconds, then, approximately 0.3 g of the sample were placed on a Walkman No.5 filter paper and a compression force of 2.5 Kg was applied for 5 minutes.


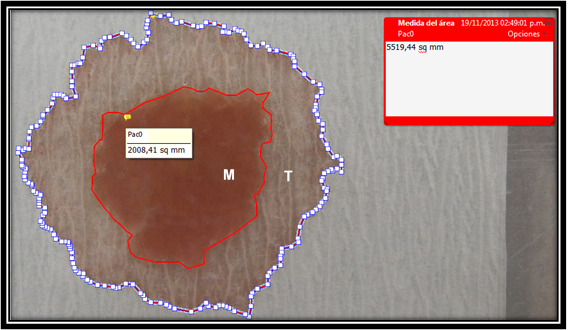


**Supplementary Figure S1b:** Then, the paper was removed, revealing two areas, one formed by the pressed meat (M) and the second corresponding to the water released by the meat (T)
