# Supplementary material for: Genome-wide association study reveals candidate genes for traits related to meat quality in Colombian Creole hair sheep
Source: Trop Anim Health Prod. 2023 Oct 12;55(6):357. doi: 10.1007/s11250-023-03688-z (PMC10570192; doi:10.1007/s11250-023-03688-z)
Supplement: Supplementary file 5 — Supplementary file5 (DOCX 51 KB) [file 11250_2023_3688_MOESM5_ESM.docx]

**SUPPLEMENTARY MATERIAL**

**Whole Genome Association Analysis (GWAS) for meat quality traits pH and CRA using a simple and multiracial linear mixed model**

Using a linear mixed model association (LMMA) with fixed effects, GWAS were performed for the three racial varieties together (OPCE, OPCS, and OPCP) and for each racial variety separately (Table 1). The criteria for selecting the best genomic association model were based on the capacity of the models to accurately detect truly associated genetic variants. Models with higher sensitivity can effectively capture the presence of genetic variants that are genuinely linked to the trait of interest. The GWAS for the overall analysis (OPCE, OPCS, and OPCP) identified 26 significant SNPs for the CRA trait and 30 SNPs for pH with a threshold of P<0.001 (Table 1). Overall, the 26 SNPs for CRA were located in 15 recognized genes in sheep. For the GWAS of pH, the 30 SNPs were located in 25 genes.

In the GWAS for each racial variety, it was found that for OPCE, 12 significant SNPs for CRA were identified (Table 1), located in 8 recognized genes in sheep. For the OPCS racial variety, 33 SNPs were identified for the CRA trait (P<0.001), located in 30 recognized genes in sheep. Finally, for OPCP, 10 SNPs for the CRA trait were identified (P<0.001, Table 3.7), located in eight recognized genes in sheep. The structural annotation of the SNPs showed that 60% were in the distal intergenic region, 30% were in introns, and 10% were in a promoter region. The GWAS analysis for pH did not yield any significant SNPs associated with the trait.

These results show that the model combining all three racial varieties exhibits a lower number of significant markers. This may be due to the non-homogeneous distribution of the breeds across regions.

Table 1. Number of significant SNPs found for GWAS of CRA and pH obtained from the linear mixed model

| **Breed** | **Modelo** | **SNPs (P<0.001) MLMA**  **CRA** | **SNPs (P<0.001) MLMA**  **pH** |
| --- | --- | --- | --- |
| Ethiopian | $y= \mu+Za+Sf+Fa+e$ | 12 | -- |
| Sudán | $y= \mu+Za+Sf+Fa+e$ | 33 | -- |
| Pelibuey | $y= \mu+Za+Sp+e$ | 10 | -- |
| Ethiopian _Sudán_Pelibuey | $y= \mu+Za+Ws+Br+Sf+Fa+e$ | 26 | -- |

| **Breed** | **Trail** | **SNP_ID** | **Chr** | **-log10(p-value)** | **Gen** | **Tipo** |
| --- | --- | --- | --- | --- | --- | --- |
| Ethiopian  _Sudán  _Pelibuey | CRA | OAR10_48722626.1 | 10 | 1,39E+00 | DIS3 | Intron |
|  |  | OAR13_7579487.1 | 13 | 4,73E+00 | TASP1 | Promotor |
|  |  | OAR13_17608488.1 | 13 | 0,57589 | LOC114117690 | Distal Intergénica |
|  |  | s53836.1 | 13 | 0.000108572 | SVIL | Intron |
|  |  | OAR14_27224601.1 | 14 | 0.000157084 | NA | Distal Intergénica |
|  |  | OAR10_13665101.1 | 10 | 0.000235593 | NA | Distal Intergénica |
|  |  | OAR15_3820697.1 | 15 | 0.000328883 | DDI1 | Distal Intergénica |
|  |  | OAR2_148273166.1 | 2 | 0.00034114 | NOSTRIN | Intron |
|  |  | OAR25_24459178.1 | 25 | 0.000368549 | NA | Intron |
|  |  | OAR10_48194802.1 | 10 | 0.000419259 | NA | Promotor |
|  |  | OAR12_26860344.1 | 12 | 0.000425511 | DUSP10 | Distal Intergénica |
|  |  | s62964.1 | 6 | 0.000443378 | NA | Intron |
|  |  | OAR15_3892898.1 | 15 | 0.000494804 | NA | Distal Intergénica |
|  |  | s52527.1 | 20 | 0.00054752 | STMND1 | Intron |
|  |  | OAR16_47146922.1 | 16 | 0.00057306 | NA | Distal Intergénica |
|  |  | OAR13_81527927.1 | 13 | 0.000578418 | NCOA3 | Intron |
|  |  | OAR1_121390041.1 | 1 | 0.000578578 | HSD17B7 | Intron |
|  |  | s08116.1 | 2 | 0.000644007 | NA | Distal Intergénica |
|  |  | OAR17_45833808.1 | 17 | 0.000646474 | NA | Distal Intergénica |
|  |  | OAR12_68590904.1 | 12 | 0.000650656 | LAMC1 | Distal Intergénica |
|  |  | s02423.1 | 4 | 0.000802579 | PCLO | Distal Intergénica |
|  |  | OAR20_37892970.1 | 20 | 0.000806858 | NA | Distal Intergénica |
|  |  | s62936.1 | 13 | 0.000850076 | MSRB2 | Intron |
|  |  | OAR25_23631385.1 | 25 | 0.000885794 | NA | Intron |
|  |  | OAR3_123918752.1 | 3 | 0.00093367 | PTPRQ | Intron |
|  |  | s47164.1 | 6 | 0.000959694 | LOC101112819 | Intron |
| **Bread** | **Trail** | **SNP_ID** | **Chr** | **p-Value** | **Gen** | **Tipo** |
| Ethiopian | CRA | OAR16_57770340.1 | 16 | 0.000155443 | - | Distal Intergénica |
|  |  | OAR16_57803053.1 | 16 | 0.000155443 | - | Distal Intergénica |
|  |  | s03399.1 | 16 | 0.000173269 | SLIT3 | Distal Intergénica |
|  |  | OAR21_54114533.1 | 21 | 0.000198044 | - | Intron |
|  |  | s36343.1 | 6 | 0.000285912 | ARAP2 | Exon |
|  |  | s35737.1 | 19 | 0.000408053 | RF00001 | Distal Intergénica |
|  |  | OAR3_96465036.1 | 3 | 0.000634918 | - | Intron |
|  |  | OAR20_48109414.1 | 20 | 0.000640164 | ELOVL2 | Distal Intergénica |
|  |  | OAR14_29971688.1 | 14 | 0.000715658 | RF00425 | Intron |
|  |  | OAR4_15640071.1 | 4 | 0.000741423 | COL28A1 | Intron |
|  |  | OAR16_7661219.1 | 16 | 0.000945693 | RF00001 | Distal Intergénica |
|  |  | s67263.1 | 3 | 0.000952077 | RF00026 | Distal Intergénica |
| **Breed** | **Trail** | **SNP_ID** | **Chr** | **p-Value** | **Gen** | **Tipo** |
| Sudán | CRA | OAR3_218139642.1 | 3 | 2,50E-02 | NA | Intron |
|  |  | s10070.1 | 9 | 2,50E-02 | RF00402 | Distal Intergénica |
|  |  | s50062.1 | 11 | 0,024963 | NA | Distal Intergénica |
|  |  | OAR17_39271762.1 | 17 | 2,50E-02 | NA | Intron |
|  |  | s17049.1 | 18 | 2,50E-02 | NA | Distal Intergénica |
|  |  | OAR24_27265846.1 | 24 | 0,024963 | KDM8 | Distal Intergénica |
|  |  | OAR5_68161647_X.1 | 5 | 1,38E-01 | NA | Distal Intergénica |
|  |  | s10696.1 | 1 | 0.000127865 | ST6GALNAC5 | Distal Intergénica |
|  |  | OAR2_151938109.1 | 2 | 0.000129957 | CSRNP3 | Distal Intergénica |
|  |  | OAR7_100406183.1 | 7 | 0.000129957 | NA | Distal Intergénica |
|  |  | OAR11_26036763.1 | 11 | 0.000184793 | AIPL1 | Distal Intergénica |
|  |  | OAR20_39711404.1 | 20 | 0.000186156 | CDKAL1 | Intron |
|  |  | OAR24_21411103.1 | 24 | 0.00023116 | LOC101102527 | Intron |
|  |  | OAR9_37599748.1 | 9 | 0.000234746 | NA | Distal Intergénica |
|  |  | OAR17_49866586.1 | 17 | 0.000311995 | ADGRD1 | Intron |
|  |  | s43108.1 | 17 | 0.000418191 | GLT1D1 | Intron |
|  |  | OAR4_59276586.1 | 4 | 0.00044205 | IFRD1 | Intron |
|  |  | OAR17_28971772.1 | 17 | 0.000445131 | LOC114110980 | Distal Intergénica |
|  |  | s65074.1 | 3 | 0.0004982 | LOC114111020 | Intron |
|  |  | OAR22_34955585_X.1 | 22 | 0.000571013 | LOC114113271 | Intron |
|  |  | OAR1_147233859.1 | 1 | 0.000571578 | LOC114114297 | Distal Intergénica |
|  |  | s52527.1 | 20 | 0.000659802 | LOC114115133 | Intron |
|  |  | OAR10_48194802.1 | 10 | 0.000683018 | LOC114114143 | Promoter |
|  |  | OAR19_39736257.1 | 19 | 0.000755601 | LOC114109775 | Intron |
|  |  | OAR15_5833133.1 | 15 | 0.000773313 | LOC114113469 | Distal Intergénica |
|  |  | s55275.1 | 13 | 0.000841183 | LOC114116250 | Distal Intergénica |
|  |  | OAR7_104475739.1 | 7 | 0.000855 | LOC114117137 | Distal Intergénica |
|  |  | OAR2_204712172.1 | 2 | 0.000922869 | BCL2L13 | Distal Intergénica |
|  |  | s10999.1 | 17 | 0.000934671 | SHOC2 | Promoter |
|  |  | OAR1_85261298.1 | 1 | 0.000952017 | LOC114111161 | Promoter |
|  |  | s07011.1 | 13 | 0.000991011 | STMND1 | Distal Intergénica |
|  |  | s24860.1 | 18 | 0.000992671 | NA | Distal Intergénica |
|  |  | s69516.1 | 20 | 0.00099807 | NA | Distal Intergenica |
|  |  | OAR15_5833133.1 | 15 | 0.000773313 | LOC114118630 | Distal Intergénica |
|  |  | s55275.1 | 13 | 0.000841183 | PRKCQ | Distal Intergénica |
|  |  | OAR7_104475739.1 | 7 | 0.000855 | NA | Distal Intergénica |
|  |  | OAR2_204712172.1 | 2 | 0.000922869 | CAVIN2 | Distal Intergénica |
|  |  | s10999.1 | 17 | 0.000934671 | NA | Promoter |
|  |  | OAR1_85261298.1 | 1 | 0.000952017 | RNPC3 | Promoter |
|  |  | s07011.1 | 13 | 0.000991011 | LOC114117690 | Distal Intergénica |
|  |  | s24860.1 | 18 | 0.000992671 | ST8SIA2 | Distal Intergénica |
|  |  | s69516.1 | 20 | 0.00099807 | NA | Distal Intergénica |
| **Breed** | **Trail** | **SNP_ID** | **Chr** | **p-Value** | **Gen** | **Tipo** |
| Pelibue | CRA | OAR2_56383269.1 | 2 | 0.000338444 | TPM2 | Promotor (<=1kb) |
|  |  | OAR13_65478113.1 | 13 | 0.000527133 | NA | Distal Intergénica |
|  |  | OAR13_69620023.1 | 13 | 0.000543749 | LOC114117623 | Promotor (1-2kb) |
|  |  | OAR13_63071015.1 | 13 | 0.000642031 | LOC114113911 | Distal Intergénica |
|  |  | s33429.1 | 20 | 0.000691486 | ATP5MD | Distal Intergénica |
|  |  | s54820.1 | 2 | 0.000742383 | CTCFL | Promotor (2-3kb) |
|  |  | OAR11_3378385.1 | 11 | 0.000776172 | LOC114109783 | Distal Intergénica |
|  |  | OAR7_102639800.1 | 7 | 0.000972862 | NANS | Distal Intergénica |
|  |  | s16895.1 | 5 | 0.00097737 | NA | Intron |
|  |  | s59858.1 | 2 | 0.000995564 | NA | Distal Intergenica |
|  |  | s16895.1 | 5 | 0.00097737 | FBN2 | Intron |
|  |  | s59858.1 | 2 | 0.000995564 | ANXA1 | Distal Intergenica |
